# The effect of CSF-1 administration on lung maturation in a mouse model of neonatal hyperoxia exposure

**DOI:** 10.1186/s12931-014-0110-5

**Published:** 2014-09-06

**Authors:** Christina V Jones, Maliha A Alikhan, Megan O’Reilly, Foula Sozo, Timothy M Williams, Richard Harding, Graham Jenkin, Sharon D Ricardo

**Affiliations:** Department of Anatomy and Developmental Biology, Monash University, Clayton, Victoria Australia; The Ritchie Centre, Monash Institute of Medical Research and Department of Obstetrics Gynaecology, Monash University, Clayton, Victoria Australia

## Abstract

**Background:**

Lung immaturity due to preterm birth is a significant complication affecting neonatal health. Despite the detrimental effects of supplemental oxygen on alveolar formation, it remains an important treatment for infants with respiratory distress. Macrophages are traditionally associated with the propagation of inflammatory insults, however increased appreciation of their diversity has revealed essential functions in development and regeneration.

**Methods:**

Macrophage regulatory cytokine Colony-Stimulating Factor-1 (CSF-1) was investigated in a model of neonatal hyperoxia exposure, with the aim of promoting macrophages associated with alveologenesis to protect/rescue lung development and function. Neonatal mice were exposed to normoxia (21% oxygen) or hyperoxia (Hyp; 65% oxygen); and administered CSF-1 (0.5 μg/g, daily × 5) or vehicle (PBS) in two treatment regimes; 1) after hyperoxia from postnatal day (P)7-11, or 2) concurrently with five days of hyperoxia from P1-5. Lung structure, function and macrophages were assessed using alveolar morphometry, barometric whole-body plethysmography and flow cytometry.

**Results and discussion:**

Seven days of hyperoxia resulted in an 18% decrease in body weight and perturbation of lung structure and function. In regime 1, growth restriction persisted in the Hyp + PBS and Hyp + CSF-1 groups, although perturbations in respiratory function were resolved by P35. CSF-1 increased CSF-1R+/F4/80+ macrophage number by 34% at P11 compared to Hyp + PBS, but was not associated with growth or lung structural rescue. In regime 2, five days of hyperoxia did not cause initial growth restriction in the Hyp + PBS and Hyp + CSF-1 groups, although body weight was decreased at P35 with CSF-1. CSF-1 was not associated with increased macrophages, or with functional perturbation in the adult. Overall, CSF-1 did not rescue the growth and lung defects associated with hyperoxia in this model; however, an increase in CSF-1R+ macrophages was not associated with an exacerbation of lung injury. The trophic functions of macrophages in lung development requires further elucidation in order to explore macrophage modulation as a strategy for promoting lung maturation.

## Introduction

Immaturity of the lungs due to preterm birth is one of the most significant complications affecting neonatal mortality. As preterm infants with respiratory distress are born with lungs at an anatomical stage not yet conducive to gas exchange, routine care practices include mechanical ventilation and oxygen supplementation. However, these lifesaving interventions can also cause permanent damage to the developing lung, resulting in a chronic lung disease termed bronchopulmonary dysplasia (BPD) [1,2]. Modern therapeutic improvements such as the administration of surfactant and corticosteroids have seen a transformation in BPD pathology away from the fibrotic injury of the past. Instead today, with many extremely preterm babies now surviving, ‘new’ BPD pathology is characterised by a disruption of alveolar formation [3,4].

Delivery of high concentrations of oxygen to neonatal animals is widely used to investigate the basis of arrested alveolar development associated with BPD in preterm infants [5,6]. In humans, alveolarisation begins *in utero* at around 36 weeks gestation, with 85% of alveoli formed after birth [7]. In the mouse, alveolar formation occurs entirely postnatally, beginning approximately 4 days after birth and proceeding until P36 [8]. Exposure of the developing lung to high levels of oxygen results in inflammation and oxidant damage, and dysregulation of alveolar development [9–11]. Understanding the mechanisms of alveolar formation has clinical relevance for the development of therapeutic strategies to improve lung maturation in preterm infants.

Macrophages are key immune cells commonly associated with inflammation and the propagation of tissue injury associated with oxygen toxicity in hyperoxia exposure. However, a greater appreciation of macrophage diversity is revealing that this heterogenous cell type is also essential in regulating organ development and regeneration [12–15]. In particular, diversity in macrophage subpopulation activation in the lung has been shown to be important in both the induction and resolution of lung injury [16].

Colony-Stimulating Factor-1 (CSF-1) is a key regulator of macrophage differentiation, proliferation, survival and activation, and acts as a pleiotropic growth factor essential in reproduction and organogenesis [17]. During pregnancy, uterine production of CSF-1 increases 1000-fold [18], and macrophages colonise the embryo and are present in large numbers in virtually all developing organs [19]. The absence of CSF-1 results in severe developmental impairment including reduced growth, neurological and reproductive defects, as well as altered development of the mammary gland, bone and pancreas [20–22]. We have previously reported that lung CSF-1R+ macrophages increase during normal lung development and peak during the alveolarisation stage [23]. In the absence of CSF-1, alveolar macrophage populations are severely depleted during postnatal development [24–26], and in adulthood mice develop spontaneous emphysema associated with deregulated matrix metalloproteinases (MMPs) and abnormal elastin deposition [26].

CSF-1 administration has been shown to enhance organogenesis. Increased branching morphogenesis was observed in embryonic kidney explants following the addition of CSF-1 [27], and in embryonic pancreas explants β-cell proliferation and insulin production were increased [28]. Furthermore, CSF-1 is suggested to preferentially regulate macrophage populations associated with trophic functions such as development [29]. While abnormalities seen in CSF-1-deficient animals are associated with a virtual absence of tissue macrophages, organogenic enhancement with CSF-1 administration is correlated with increased macrophage numbers [20–22,27,28].

CSF-1-responsive macrophages in developing embryonic kidneys and lungs exhibit a gene expression profile characteristic of an M2 or tissue remodelling-type macrophage [27]. Similarly we have demonstrated the importance of trophic macrophages in alveolarisation, with an upregulation of M2 genes observed during this developmental stage in the mouse [23]. Furthermore, CSF-1 has important immunomodulatory properties, and in a murine model of neonatal hyperoxia, mesenchymal stem cell (MSC)-mediated amelioration of injury was associated with increased CSF-1 levels [30]. We hypothesise that the administration of CSF-1 to promote augmentation of developmental macrophages associated with alveolarisation may reduce the need for damaging long-term oxygen therapy in preterm newborns and attenuate the developmental arrest of the lung associated with BPD.

The aim of this study was to determine the impact of CSF-1 administration on lung maturation in a model of hyperoxia-associated lung developmental perturbation, when delivered either as a treatment after hyperoxia or when administered prophylactically with concurrent hyperoxia exposure. In particular, we assessed lung functional maturation and the impact of a CSF-1-mediated increase in macrophages. We found that CSF-1 did not rescue the growth and pulmonary defects associated with hyperoxia in this model; however the concomitant increased infiltration of CSF-1-responsive macrophages was not associated with exacerbation of lung injury.

## Methods

### Hyperoxia exposure

All animal experiments were approved by the Monash University Animal Ethics Committee and conducted in accordance with the “Australian Code of Practice for the Care and Use of Animals for Scientific Purposes”. This study utilised *csf1r*-EGFP mice, which express enhanced green fluorescent protein (EGFP) in cells of the myeloid lineage via the insertion of an EGFP transgene under the control of the *Csf1r* promoter [31]. Pregnant *csf1r*-EGFP mice were allowed to litter-down naturally in individually ventilated cages (Green Line IVC Sealsafe; Techniplast, Buguggiate, Italy). Litters were randomly assigned to receive either normoxia (ambient room air; 21% oxygen) or hyperoxia (65% oxygen). Hyperoxia was achieved by mixing 100% medical grade oxygen and medical grade dry air, delivered to cages via Perspex(R) tubing into ventilation ports. Oxygen and carbon dioxide concentrations were continuously monitored by a gas analyser (Servoflex MiniMP 5200, Servomex, Valley Point, Singapore). Dams remained with their own pups for the entire experiment. Boxes were briefly disconnected from hyperoxia gases for a maximum of 15 minutes when injections were performed. Following hyperoxia exposure (regime 1: 7 days; regime 2: 5 days), mice were maintained in room air until experimental endpoints at P11 and P35.

### CSF-1 administration

Hyperoxia-exposed littermate pups received either mouse recombinant CSF-1 (0.5 μg/g body weight; University of Queensland Protein Facility, Brisbane, Australia) or vehicle (PBS), administered via intraperitoneal (i.p.) injection at a final volume of 25 μl. Biological activity was confirmed with *in vitro* culture assays demonstrating CSF-1-mediated proliferation of bone-marrow derived macrophages (data not shown). In treatment regime 1, neonatal pups were continuously exposed to hyperoxia from birth for 7 days with subsequent injections performed once daily for 5 days from P7-11. In treatment regime 2, newborn pups were exposed to hyperoxia and were administered CSF-1 concurrently, and therefore a shorter exposure of 5 days was used to facilitate 5 concurrent daily injections. At experimental endpoints (regime 1: P11 and P35; regime 2: P5 and P35) mice were humanely euthanised via cervical dislocation for morphometric or flow cytometric analysis.

### Plethysmography

Respiratory function was assessed using unrestrained barometric whole-body plethysmography, as described previously [23,32,33]. In brief, mice were placed in a sealed cylindrical Perspex chamber (Neonate; 75 mm × 50 mm, Adolescent/Adult; 150 mm × 50 mm), and changes in pressure caused by tidal breathing movements were measured using a volumetric pressure transducer (model PT5A; Grass Instrument Co., Quincy, MA, USA) and amplified (Octal Bridge Amp model ML228 and Powerlab 8/30 model ML870; ADInstruments, Bella Vista, NSW, Australia). Pressure fluctuations were recorded using Chart™ software (v5.1; ADInstruments). At the beginning of each session the plethysmograph was calibrated by measuring the pressure deflection caused by the injection of a known volume (300 μl) of air into the chamber. The temperature and relative humidity within the chamber were noted at the beginning and end of recordings (model HM34; Vaisala, Hawthorn, VIC, Australia). Waveform analysis (Chart™; ADInstruments) was used to derive breath frequency (breaths/min), total breath cycle time (ms), inspiration time (ms), expiration time (ms), tidal volume (μL), minute volume (mL/min), inspiratory duty cycle (%) and inspiratory flow rate (μL/sec). Measurements were performed at P5 or P7 at the end of hyperoxia, following CSF-1 administration at P11 and then at P14, P21, P28 and P35.

### Morphometry

For morphometric assessments, the trachea was cannulated immediately after euthanasia and the lungs inflation-fixed *in situ* via intratracheal instillation of 10% buffered formalin at a pressure of 20 cmH_2_0. The entire thorax was subsequently immersion fixed for 24 hr before lungs were dissected. The left lobe was processed, embedded in paraffin wax, sectioned at 5 μm, mounted on Polysine™ slides (Menzel-Glaser, Braunschweig, Germany) and stained with haematoxylin and eosin. Images from 15 non-overlapping fields of view were captured at × 400 magnification and ImagePro Plus software (Media Cybernetics, Bethesda, MA, USA) was used to assess mean linear intercept (MLI), as an estimate of alveolar diameter, and tissue/airspace percentage, as described previously [34]. In brief, MLI was calculated by superimposing two transverse lines over lung images and counting intersecting points. The percentage airspace and tissue was estimated by superimposing 21 equidistant line segments over lung images and counting line termini intersections with either tissue or airspace, respectively. Fields of view containing parenchyma only were analysed, with care taken to exclude areas with airways or extensive vasculature. Measurements were conducted in a blinded manner by the same individual (CVJ) to reduce bias.

### Flow cytometry

To assess macrophage populations in hyperoxic lungs and their response to CSF-1 treatment, whole lungs of littermate mice underwent flow cytometric analysis at the end of treatment at P11 or P5, with the final injection being administered 3 hours before lungs were collected. Whole lungs underwent enzymatic and mechanical digestion to yield a single cell suspension as described previously [12]. Red blood cells were lysed and cell suspensions were filtered through a 40 μm cell strainer (BD Biosciences, North Ryde, NSW, Australia). Cell counts were performed using a Coulter® Particle Count and Size Analyzer (Beckman Coulter Australia Pty Ltd, Gladesville, NSW, Australia). 3 × 10^6^ cells were immunolabelled with anti-CD45 APC Cy7-conjugated (1:800; BioLegend, San Diego, CA, USA; Clone 30-F11) and anti-F4/80 APC-conjugated (1:200; eBioscience, San Diego, CA, USA; Clone BM8) antibodies. Samples were run on a BD FACSCanto II cytometer (BD Biosciences) and data analysis was performed using FlowJo FCS analysis software (Tree Star Inc., Ashland, OR, USA).

### Statistical analysis

This study included 17 litters of mice, ranging between 4 and 8 pups per litter. The N numbers for bodyweight and lung function analysis outlined in appropriate figure legends are a cumulative of a minimum two repeated experiments. Flow cytometric analysis was performed on one representative litter for inter-litter comparison. Data are presented as mean ± standard error of the mean (SEM). Graphing and statistical analyses were performed using GraphPad Prism™ (GraphPad Software; Section 3.14). Specific statistical tests used are outlined in figure legends. A p value of less than 0.05 was considered statistically significant.

## Results

### Neonatal hyperoxia exposure for 7 days results in decreased body weight and perturbations in lung structure and function

**Figure 1:**
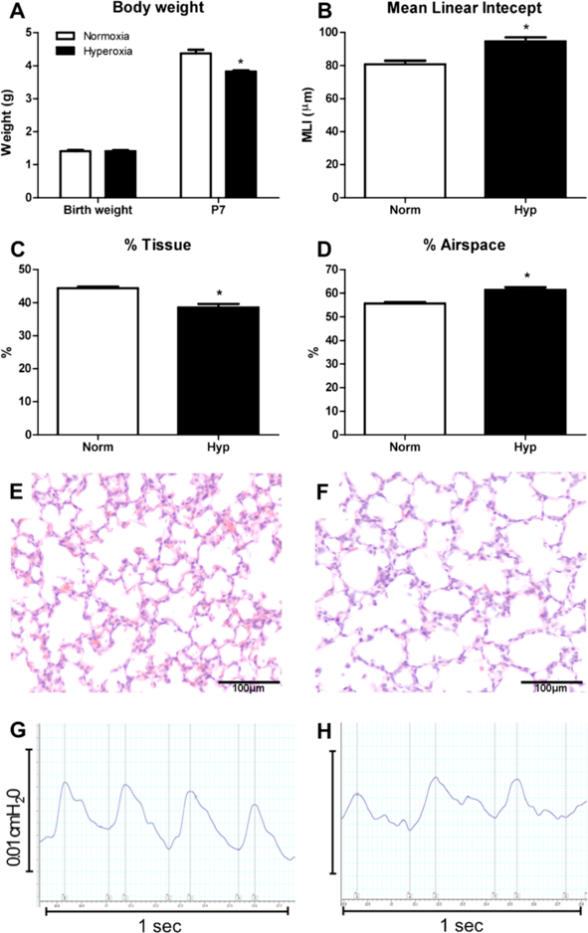
**Neonatal hyperoxia exposure resulted in decreased growth and lung structural and functional alterations.** Body weights of mice exposed to hyperoxia for 7 days from birth compared to neonates exposed to normoxia **(A)**. n = 9 & 13 mice/treatment. Morphometric estimation of mean linear intercept **(B)**, percentage tissue **(C)** and percentage airspace **(D)** in lungs exposed to hyperoxia and normoxia. n = 4 & 5 lungs/treatment. Data were analysed using two-tailed unpaired t-tests. *p < 0.05. Normoxia **(E)** and hyperoxia-exposed **(F)** lungs at P7 demonstrated the decrease in tissue percentage and increase in alveolar size due to hyperoxia. Respiratory trace recordings revealed that mice exposed to hyperoxia **(H)** display abnormal breath patterns compared to normoxic mice **(G)**.

Neonatal murine hyperoxia is a model of impaired alveologenesis and a clinically relevant model of preterm lung damage. In our hyperoxia model, neonatal mice were exposed to 65% oxygen from birth for 7 days. Hyperoxia significantly decreased growth of neonatal mice. At birth there was no significant difference in body weight between groups (p = 0.09); however after 7 days of hyperoxia exposure body weight was decreased by 18% compared to normoxia-exposed offspring (4.37 ± 0.12 g vs. 3.57 ± 0.01 g, p < 0.001; Figure 1A). Lung structural alterations were assessed via morphometric analyses. Compared to normoxia, hyperoxia resulted in a 17% increase in MLI (80.9 ± 2.1 μm vs. 94.6 ± 2.4 μm, p < 0.05; Figure 1B), a 6% decrease in percentage tissue (44.3 ± 0.5% vs. 38.6 ± 1.1%, p < 0.05; Figure 1C) and a corresponding 6% increase in percentage airspace (55.7 ± 0.5% vs. 61.4 ± 1.1%, p < 0.05; Figure 1D). These quantitative measurements reflect the histological changes observed in hyperoxic lungs, where alveoli appeared larger and secondary septation appeared to be impaired (Figure 1F), in comparison to the lungs of normoxic mice which had smaller alveoli and evidence of extensive secondary septation (Figure 1E). These structural perturbations associated with hyperoxia exposure were reflected in functional alterations (Table 1). Unrestrained barometric whole-body plethysmography assessment of respiratory function after 7 days of hyperoxia indicated that these mice exhibited abnormal breathing patterns compared to normoxic mice (Figure 1G&H). Quantification revealed significant reductions in tidal volume (21% p < 0.0001), minute volume (29% p < 0.01), breath frequency (9% p < 0.05) and inspiratory flow rate (30% p < 0.001; Table 1). Functional alterations also included significant increases in total cycle time (9% p < 0.05), expiration time (50% p < 0.001) and a trend towards increased inspiration time (14% p = 0.08).

**Table 1:** **Effect of neonatal hyperoxia exposure on lung function**

	**Normoxia**	**Hyperoxia**	**Significance**	**p value**
**Tidal volume** (μl)	13.64 ± 0.76	10.74 ± 0.34	***	*0.0008*
**Minute volume** (ml/min)	3.28 ± 0.25	2.34 ± 0.13	**	*0.0014*
**Frequency** (breaths/min)	238.0 + 6.63	216.4 ± 6.18	*	*0.031*
**Total cycle time** (ms)	254.2 ± 7.32	276.9 ± 7.07	*	*0.044*
**Inspiration time** (ms)	72.51 ± 3.85	82.75 ± 3.64		*0.076*
**Expiration time** (ms)	137.7 ± 22.11	206.4 ± 10.76	**	*0.005*
**Inspiratory flow rate** (μl/sec)	191.6 ± 12.93	133.5 ± 8.06	***	*0.0006*
**Inspiratory duty cycle** (%)	28.73 ± 1.66	29.99 ± 1.29		0.55
**Tidal volume** (μl)/**bw** (g)	3.11 ± 0.11	2.98 ± 0.17		0.56
**Minute volume** (ml/min)/**bw** (g)	744.0 ± 42.11	650.8 ± 48.17		0.19

*p < 0.05, **p < 0.01, ***p <0.001.

Plethysmographic analysis of respiratory parameters in neonates exposed to normoxia or hyperoxia for 7 days following birth. Hyperoxia exposure resulted in significant perturbation of lung function at P7, compared to normoxia controls, n = 9/16 per group. Data were analysed using two-tailed unpaired t-tests. Bw – body weight.

### CSF-1 treatment after hyperoxia did not rescue body weight, but increased macrophage numbers were not associated with exacerbation of lung structural or functional perturbation

**Figure 2:**
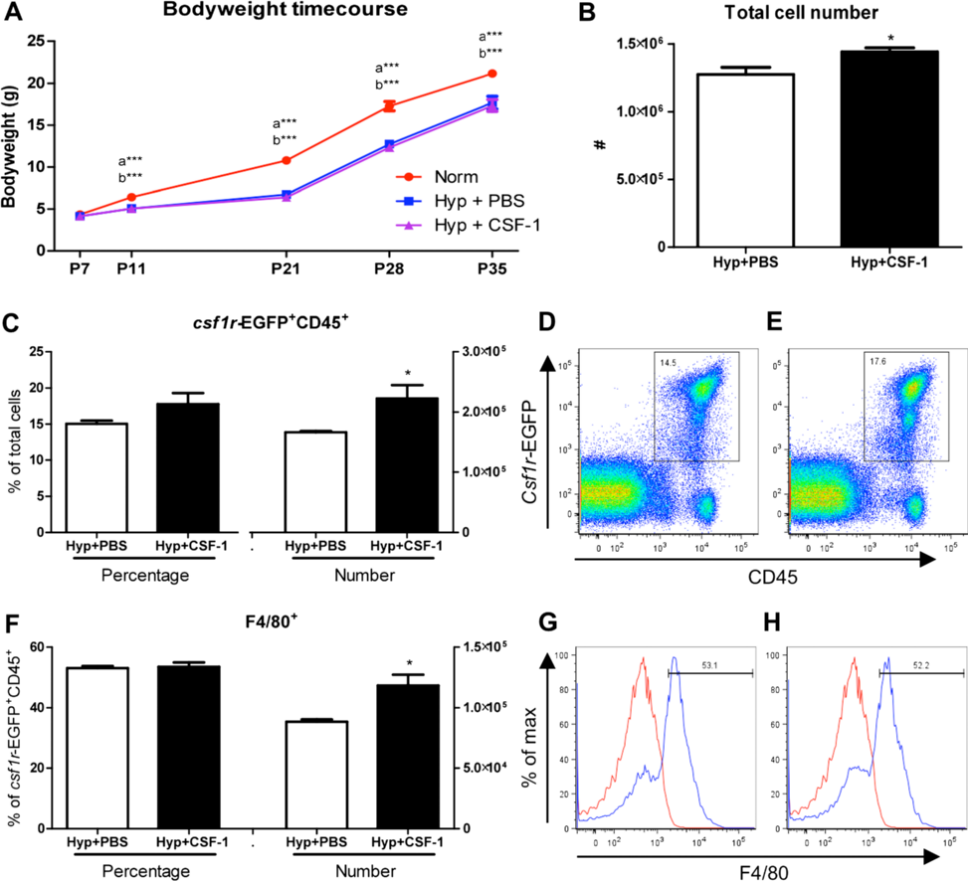
**Growth and macrophage analysis of mice treated with CSF-1 following hyperoxia.** Body weights of mice treated with CSF-1 (Hyp + CSF-1; purple line) or PBS (Hyp + PBS; blue line) following hyperoxia exposure was significantly decreased compared to normoxic mice (Norm; red line) **(A)**. n = 4-13 mice/time point. Data were analysed using one-way ANOVAs and Tukey’s post-hoc tests. ‘a’ represents significant difference between Norm and Hyp + PBS, ‘b’ represents significant difference between Norm and Hyp + CSF-1. Flow cytometric analysis of littermate lungs at P11 treated with CSF-1 or PBS controls following hyperoxia **(B-H)**. Total lung cells **(B)** were gated on *csf1r*-EGFP+ CD45+ cells to quantitate CSF-1R+ cell number and proportion **(C)** after CSF-1 **(D)** and PBS treatment **(E)**. F4/80 expression demarcates mature macrophages within the CSF-1R+ population **(F-H)**. Histograms revealing gating procedure for F4/80 expression in representative PBS **(G)** and CSF-1-treated lungs **(H)**. Staining (blue) is overlayed with an isotype control (red). CSF-1 treatment resulted in a significant increase in total cellularity, CSF-1R+ cell number and macrophage number. n = 4 littermate lungs/treatment. Data were analysed using two-tailed unpaired t-tests. *p < 0.05, ***p < 0.001.

In the first treatment regime, CSF-1 was administered after 7 days of hyperoxia-induced lung injury to assess the ability of CSF-1 therapy to rescue growth and promote alveolar development. Comparing hyperoxia and normoxia groups, a significant reduction in body weight was observed in Hyp + PBS mice, which persisted from P11 (6.43 ± 0.34 g vs. 5.07 ± 0.13 g, p < 0.001) to adulthood at P35 (21.17 ± 0.47 g vs. 17.70 ± 0.76 g, p < 0.001; Figure 2A). While both groups exposed to hyperoxia weighed significantly less than the Norm group, there was no significant difference in growth between the Hyp + PBS and Hyp + CSF-1 groups (Figure 2A). From P11 at the end of treatment (5.07 ± 0.13 g vs. 5.05 ± 0.16 g) to adulthood at P35 (17.70 ± 0.76 g vs. 17.35 ± 0.70 g), CSF-1 treatment did not rescue body weight when compared to mice exposed to hyperoxia and administered PBS (Figure 2A).

Removal of mice from hyperoxia resulted in a marked improvement in lung structure. At the end of treatment at P11, there was no significant difference in MLI, percentage tissue or percentage airspace amongst any of the three groups (Table 2). However, there was a trend towards increased MLI in the Hyp + PBS group compared to Norm, and this increase was not observed in the CSF-1 treated group. In adulthood at P35, no significant differences were observed in any of the lung structure parameters investigated in any group. However, of note is that all Hyp + CSF-1 values were more similar to Norm values than Hyp + PBS, indicating that structural abnormalities had not been exacerbated with CSF-1 treatment.

**Table 2:** **Morphometric analysis of lungs from CSF-1-treated mice after 7 days of neonatal hyperoxia exposure**

		**Normoxia**	**Hyp + PBS**	**Hyp + CSF-1**
**P11**	**% Tissue**	50.66 ± 1.61	53.62 ± 2.99	52.98 ± 1.90
	**% Airspace**	49.34 ± 1.61	46.38 ± 2.99	47.02 ± 1.90
	**MLI**	88.59 ± 2.42	94.33 ± 2.37	87.42 ± 0.99
**P35**	**% Tissue**	36.97 ± 1.29	39.15 ± 2.01	35.92 ± 0.87
	**% Airspace**	63.03 ± 1.29	60.85 ± 2.01	64.08 ± 0.87
	**MLI**	64.99 ± 1.78	68.67 ± 1.64	67.34 ± 1.63

Morphometric estimation of percentage tissue, percentage airspace and mean linear intercept (MLI) at P11 (n = 4 lungs/treatment) and P35 (n = 6-7 lungs/treatment) in newborn mice exposed to normoxia or hyperoxia for 7 days followed by either CSF-1 or PBS treatment for 5 consecutive days. No significant changes were observed in the morphometric parameters examined amongst the three treatment groups. Data were analysed using one-way ANOVAs and Tukey’s post-hoc tests.

**Figure 3:**
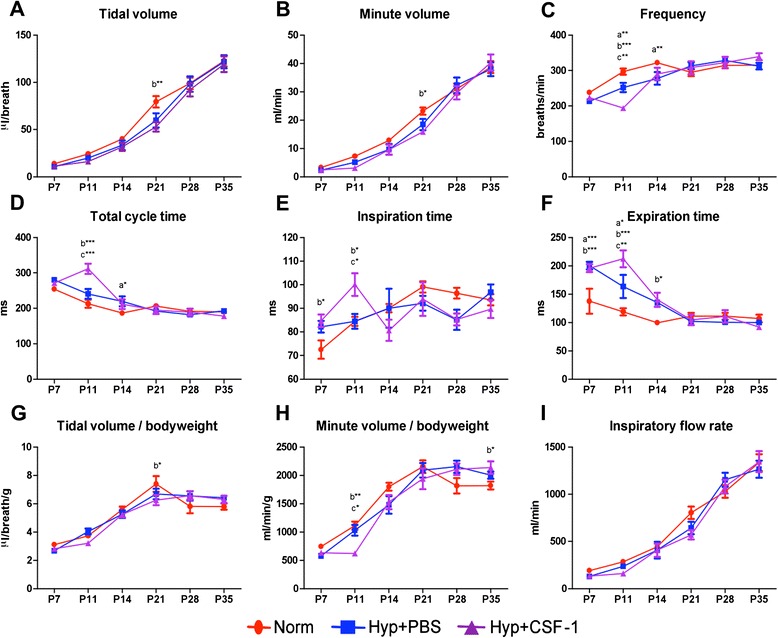
**Lung function of mice treated with CSF-1 following hyperoxia exposure.** Plethysmography assessment of lung function parameters - tidal volume **(A)**, minute volume **(B)**, frequency **(C)**, total cycle time **(D)**, inspiration time **(E)**, expiration time **(F)**, tidal volume/bodyweight **(G)**, minute volume/bodyweight **(H)**, inspiratory flow rate **(I)** - in mice treated with CSF-1 (Hyp + CSF-1; purple line) or PBS (Hyp + PBS; blue line) following hyperoxia exposure, compared to normoxic controls (Norm; red line). n = 6-14 mice/treatment/time point. Data were analysed using two-way ANOVAs and Bonferonni post-hoc tests. ‘a’ represents significant difference between Norm and Hyp + PBS, ‘b’ represents significant difference between Norm and Hyp + CSF-1, ‘c’ represents significant difference between Hyp + PBS and Hyp + CSF-1. *p < 0.05, **p < 0.01, ***p < 0.001.

A time course analysis of lung function was performed from the conclusion of hyperoxia exposure at P7, at the end of treatment at P11, and weekly throughout development from P14 to adulthood at P35 (Figure 3). Tidal volume (Figure 3A), minute volume (Figure 3B), tidal volume/body weight ratio (Figure 3G) and inspiratory flow rate (Figure 3I) all increased during postnatal development, with all three groups following a similar trajectory. No significant differences were observed at any time point, with the exception of P21 when a significant reduction in tidal volume (79.5 ± 6.0 μl vs. 53.0 ± 5.0 μl, p < 0.01; Figure 3A), minute volume (23.2 ± 1.3 ml vs. 15.9 ± 0.8 ml, p < 0.05; Figure 3B) and tidal volume/body weight (7.4 ± 0.6 μl/g vs. 6.3 ± 0.4 μl/g, p < 0.05; Figure 3G) was observed in the Hyp + CSF-1 group compared to Norm. Some early alterations were observed in parameters including frequency (Figure 3C), total cycle time (Figure 3D), inspiration time (Figure 3E) and minute volume/body weight (Figure 3H), where at P11 CSF-1-treated mice had significant alterations in breathing patterns compared to Norm or Hyp + PBS mice. Furthermore, only in frequency (Figure 3C), expiration time (Figure 3F) and minute volume/body weight (Figure 3H) was there an alteration with CSF-1 treatment that was significantly different compared to PBS administration at P11. However, any earlier perturbations associated with hyperoxia exposure and/or CSF-1 administration resolved and at P35 no significant difference was observed amongst the three treatment groups in the breathing parameters examined.

To investigate the impact of CSF-1 treatment on lung macrophages, flow cytometry was performed on littermate hyperoxic lungs after treatment with either PBS or CSF-1 at P11 (Figure 2). The total lung cell number was significantly increased by 13% in mice treated with CSF-1 (128 ± 5.3 × 10^5^ vs. 144 ± 3.1 × 10^5^, p < 0.05; Figure 2B). Transgenic *csf1r*-EGFP mice, which express EGFP under control of the *csf1r* promoter, identified CSF-1-responsive cells. The number of CSF-1R+ cells significantly increased by 34% with CSF-1 treatment compared to PBS (16.7 ± 0.2 × 10^4^ vs. 22.2 ± 2.2 × 10^4^, p < 0.05; Figure 2C). CSF-1R was not expressed outside the leukocyte population, with no CD45- lung cells found to express the EGFP transgene (Figure 2D&E). F4/80 expression was used to identify mature macrophages (Figure 2F-H). The number of F4/80+ macrophages significantly increased by 34% with CSF-1 treatment at P11 (88 ± 2.0 × 10^3^ vs. 118 ± 9.0 × 10^3^, p < 0.05; Figure 2F).

### Delivery of CSF-1 in conjunction with hyperoxia exposure resulted in reduced body weight; however structural alterations were not exacerbated and pulmonary macrophages were not increased

**Figure 4:**
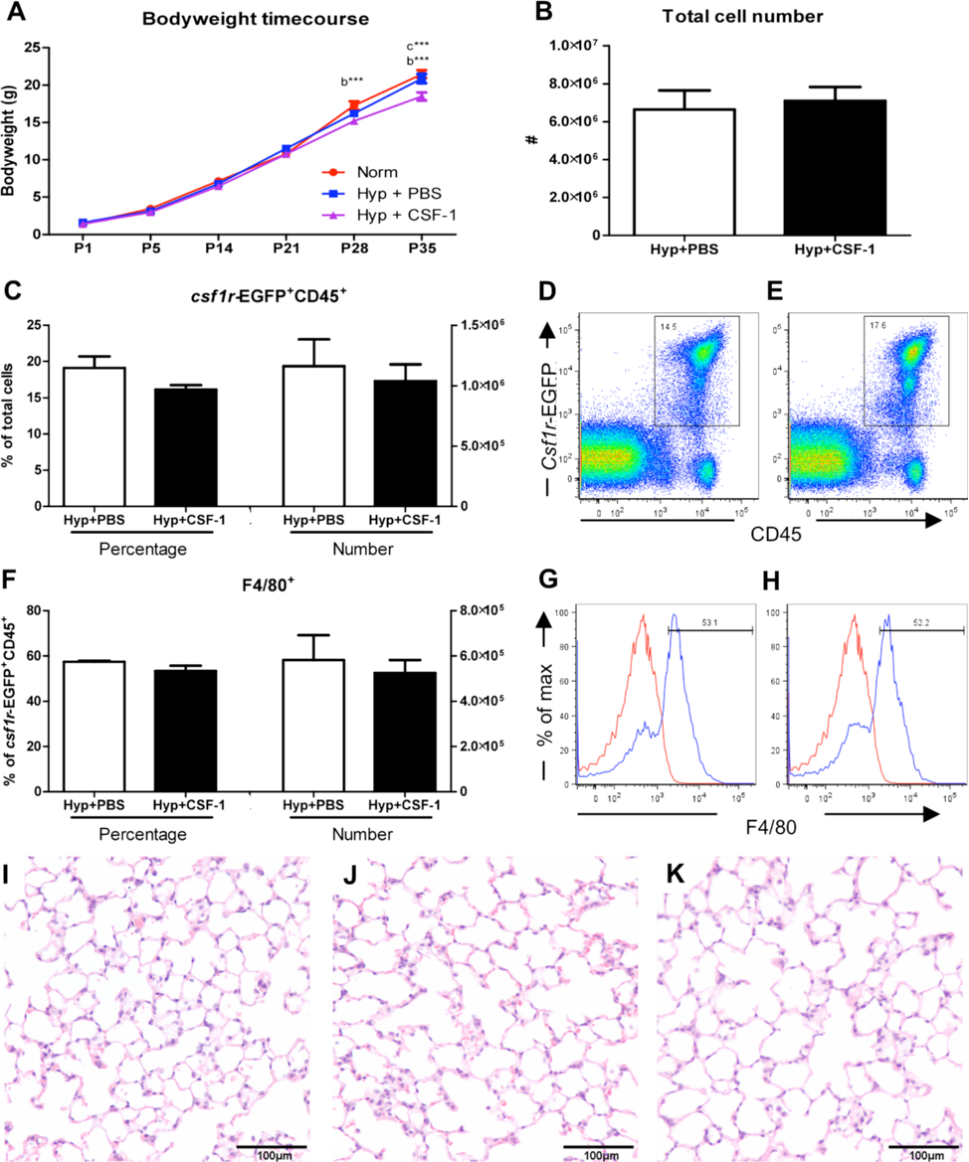
**Growth and macrophage analysis in mice treated with CSF-1 during concurrent hyperoxia exposure.** Body weights of mice treated with CSF-1 (Hyp + CSF-1; purple line) or PBS (Hyp + PBS; blue line) during hyperoxia exposure compared to normoxic mice (Norm; red line) **(A)**. n = 4-13 mice/time point. Data were analysed using a two-way ANOVA and Bonferonni post-hoc test. ‘b’ represents significant difference between Norm and Hyp + CSF-1, ‘c’ represents significant difference between Hyp + PBS and Hyp + CSF-1. ***p < 0.001. Flow cytometric analysis of littermate lungs at P5 following treatment with CSF-1 or PBS during hyperoxia. Total lung cells **(B)** were gated on *csf1r*-EGFP+ CD45+ cells to quantitate CSF-1R+ cell number and proportion **(C)** after CSF-1 **(E)** and PBS treatment **(D)**. F4/80 expression demarcates mature macrophages within the CSF-1R+ population **(F-H)**. Histograms revealing gating procedure for F4/80 expression in representative PBS **(G)** and CSF-1-treated lungs **(H)**. Staining (blue) is overlayed with an isotype control (red). n = 3 littermate lungs/treatment. Data were analysed using two-tailed unpaired t-tests. Photomicrographs of Norm **(I)**, Hyp + PBS **(J)** and Hyp + CSF-1 **(K)** lungs at P35 stained with H&E demonstrated fewer, larger alveoli with hyperoxia exposure **(J & K)**.

In the second treatment regime, CSF-1 was administered in conjunction with high oxygen exposure to assess the potential of CSF-1 to mitigate lung damage and promote alveolar formation. In this more clinically relevant regime, neonatal mice were exposed to hyperoxia for 5 days to allow for 5 concurrent daily injections of CSF-1 or PBS. Body weight was measured to assess growth over the time course of development, from the end of treatment at P5, and weekly throughout postnatal development from P14 to adulthood at P35 (Figure 4A). Body weight was not significantly altered in the Hyp + PBS group compared to Norm. These two treatment groups followed a similar growth trajectory and no significant changes were observed at any of the time points examined. However, Hyp + CSF-1 was found to negatively affect body weight, although this emerged only at later time points. By P28, the growth trajectory was decreased in the Hyp + CSF-1 group and body weight was significantly lower than in the Norm group (17.27 ± 0.54 g vs. 15.20 ± 0.42 g, p < 0.001). Also, at P35, body weight was significantly lower in the Hyp + CSF-1 group compared to both the Norm (21.47 ± 0.51 g vs. 18.50 ± 0.53 g, p < 0.001) and Hyp + PBS groups (20.89 ± 0.61 g vs. 18.50 ± 0.53 g, p < 0.001; Figure 4A).

The effect of hyperoxia and concurrent CSF-1 treatment on structural development of the lung was assessed at P5 (Table 3). No significant differences were observed in the structural parameters examined between the three groups. A trend of altered structural measurements was observed in the Hyp + PBS group compared to Norm, although it did not reach significance. In the Hyp + CSF-1 group, all parameters measured approached the values of the Norm group and structural alterations were not exacerbated with CSF-1 administration. In the adult at P35, percentage tissue and airspace were unaltered in the Hyp + PBS or Hyp + CSF-1 groups compared to Norm. However, MLI was affected by hyperoxia exposure. Compared to Norm, a significant increase in MLI was observed in the Hyp + PBS (65.0 ± 1.8 μm vs. 72.7 ± 1.2 μm, p < 0.05) and the Hyp + CSF-1 groups (65.0 ± 1.8 μm vs. 75.3 ± 2.8 μm, p < 0.01; Table 3). CSF-1 treatment did not significantly affect the outcome of hyperoxia on the lung, as no significant difference in MLI compared to PBS was observed. Histologically, there were fewer and larger alveoli in the lungs of mice exposed to both Hyp + PBS (Figure 4J) and Hyp + CSF-1 (Figure 4K) as compared to Norm controls (Figure 4I).

**Table 3:** **Morphometric analysis of lungs from mice administered CSF-1 during concurrent hyperoxia exposure**

		**Normoxia**	**Hyp + PBS**	**Hyp + CSF-1**
**P5**	**% Tissue**	34.92 ± 1.83	28.31 ± 1.83	32.15 ± 1.74
	**% Airspace**	65.08 ± 1.83	71.69 ± 1.83	67.85 ± 1.74
	**MLI**	113.00 ± 3.22	121.70 ± 7.97	116.40 ± 3.45
**P35**	**% Tissue**	36.97 ± 1.29	38.48 ± 2.01	36.92 ± 0.88
	**% Airspace**	63.03 ± 1.29	61.52 ± 2.01	63.08 ± 0.88
	**MLI**	64.99 ± 1.78	72.66 ± 1.22 a*	75.27 ± 2.79 b**

Morphometric estimation of percentage tissue, percentage airspace and mean linear intercept at P5 at the cessation of treatment (n = 4-5/group), and at P35 (n = 6-7/group). Data were analysed by one-way ANOVA and Tukey’s post-hoc test. ‘a’ represents significant difference between Norm and Hyp + PBS, ‘b’ represents significant difference between Norm and Hyp + CSF-1. *p < 0.05, **p < 0.01.

**Figure 5:**
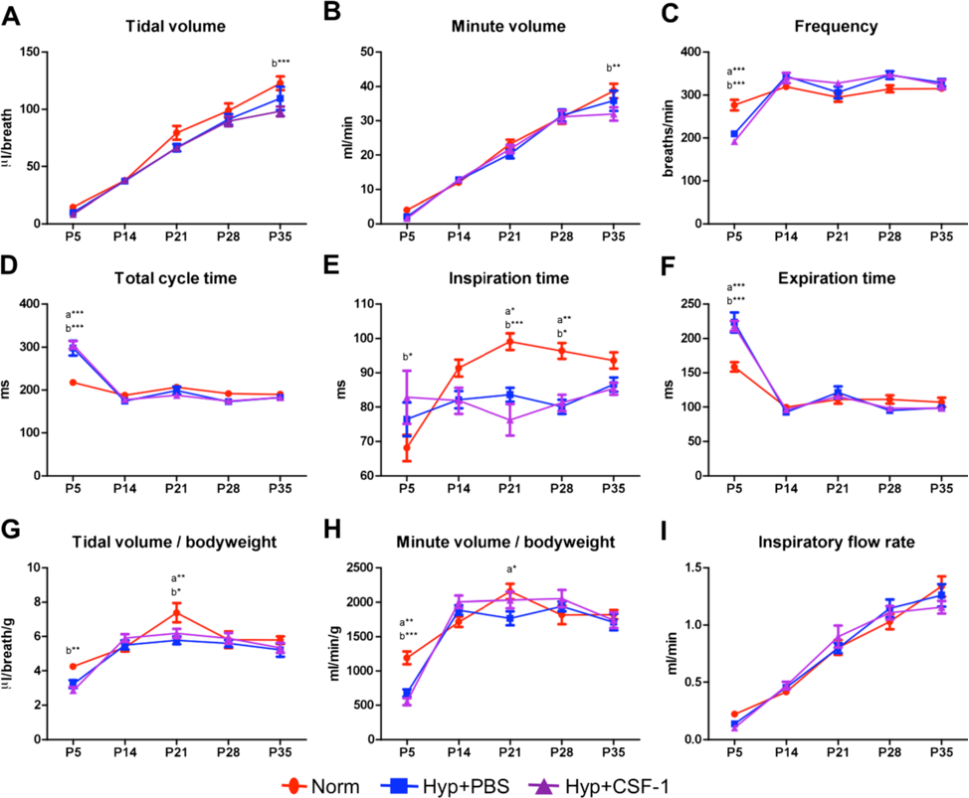
**Lung function of mice treated with CSF-1 during concurrent hyperoxia exposure.** Plethysmographic assessment of lung function parameters - tidal volume **(A)**, minute volume **(B)**, frequency **(C)**, total cycle time **(D)**, inspiration time **(E)**, expiration time **(F)**, tidal volume/bodyweight **(G)**, minute volume/bodyweight **(H)**, inspiratory flow rate **(I)** - in mice treated with CSF-1 (Hyp + CSF-1; purple line) or PBS (Hyp + PBS; blue line) during hyperoxia exposure compared to normoxia (Norm; red line). n = 6-14 mice/treatment/time point. Data were analysed using two-way ANOVAs and Bonferonni post-hoc tests. ‘a’ represents significant difference between Norm and Hyp + PBS, ‘b’ represents significant difference between Norm and Hyp + CSF-1. *p < 0.05, **p < 0.01, ***p < 0.001.

Plethysmographic analysis of breath parameters (Figure 5) indicated that initial perturbations at P5 were present in breath frequency (Figure 5C), total cycle time (Figure 5D), inspiration time (Figure 5E), expiration time (Figure 5F) and minute volume/body weight ratio (Figure 5H). However, these alterations were observed in both hyperoxia groups compared to Norm, and were thus not a result of CSF-1 delivery. After initial perturbation at P5, breathing frequency, total cycle time and expiration time were subsequently comparable amongst all treatment groups. Inspiration time was altered in the hyperoxia groups compared to Norm, with a significant decrease at P21 (Norm vs. Hyp + PBS 15%, p < 0.05; Norm vs. Hyp + CSF-1 23%, p < 0.01) and P28 (Norm vs. Hyp + PBS 17%, p < 0.01; Norm vs. Hyp + CSF-1 16%, p < 0.05; Figure 5E). Tidal volume (Figure 5A), minute volume (Figure 5B) and inspiratory flow rate (Figure 5I) increased during postnatal life and followed a similar trajectory in all three treatment groups. No significant differences were observed in inspiratory flow rate at any time points. However, at P35 in the Hyp + CSF-1 group there was a significant decrease in tidal volume (122.84 ± 5.98 μl vs. 98.36 ± 4.26 μl, p < 0.001; Figure 5A) and minute volume (38.7 ± 2.1 ml vs. 32.0 ± 1.9 ml, p < 0.01; Figure 5B) compared to Norm.

To investigate the impact of CSF-1 treatment on lung macrophages, flow cytometry was performed on littermate lungs at P5, after either PBS or CSF-1 treatment was delivered concurrently with hyperoxia. Total lung cell number was not altered with CSF-1 treatment, with no significant change compared to PBS was observed (Figure 4B). *Csf1r*-EGFP transgene expression on CD45+ leukocytes revealed that the number and percentage of CSF-1R+ cells within the lung was comparable amongst both PBS and CSF-1-treated mice (Figure 4C-E). Similarly, when cells were further gated on F4/80 expression to identify macrophages (Figure 4G&H), no change in the number or percentage of macrophages in the lung was observed as a result of CSF-1 treatment (Figure 4F).

## Discussion

Oxygen supplementation remains a critical and lifesaving intervention for infants with respiratory distress; however, it can result in detrimental alterations to the developing lung. Exposing neonatal mice to high concentrations of oxygen mimics the damage to the lung associated with BPD, providing a relevant model to assess the function of CSF-1 in rescuing and enhancing alveolar formation. This study employed a hyperoxia regime of 65% oxygen for 1 week after birth, providing a milder, more clinically-relevant exposure that more closely resembles the oxygen therapy regime of ventilated premature infants than other previous studies [5,35]. It was also selected to minimize maternal oxygen toxicity and avoid stress and abandonment issues associated with dam rotation. Hallmarks of this hyperoxia model included growth restriction, impaired lung structural development indicative of reduced alveolarisation, and as a consequence measurable perturbations of respiratory function. While body weight was comparable at birth, hyperoxia impacted negatively on neonatal growth as evidenced by the reduction in body weight in the hyperoxia group by the end of exposure. Although there is potential for maternal oxygen toxicity to impact on the growth restriction observed, such growth restriction associated with this model is important to examine the growth promoting functions of CSF-1 as observed previously [23]. Lung structural alterations characteristic of oxygen toxicity and BPD were also observed. MLI and percentage airspace were increased, while percentage tissue was decreased. This is in agreement with other hyperoxia studies, where oxygen supplementation caused BPD-like pathological changes to the lung parenchyma and impaired alveolarisation resulting in fewer, larger alveoli [5,6].

Functional perturbations were also revealed following hyperoxia. Unrestrained barometric whole-body plethysmography proved a beneficial analytical technique sensitive enough to reveal measurable differences in neonates at 7 days of age. While the accuracy of absolute quantitation in small animals is debatable [36], this technique provides a qualitative comparative assessment applicable to this study. Furthermore, the non-invasive nature of this technique makes it invaluable for these experiments in assessing the effects of CSF-1 and the rescue of lung function over time. Abnormal breathing patterns were associated with hyperoxia exposure after 7 days. In agreement with previously reported effects of hyperoxia on pulmonary activity [37], changes included an increase in total cycle time primarily due to lengthened expiratory time. Furthermore, hyperoxia resulted in a reduction in tidal volume, supporting the structural observation of impaired alveolar formation. However, effects on tidal and minute volumes were mitigated when normalised to body weight, suggesting that the overall growth restriction contributes to the reduced lung volumes.

Harnessing the organogenic importance of growth factors involved in alveolar formation has been a fundamental strategy for promoting lung maturation. Factors such as keratinocyte growth factor [38], hepatocyte growth factor [39], retinoic acid [40] and vascular endothelial growth factor [41] are important in alveolarisation and have shown positive effects in rescuing alveolar development in hyperoxic animal studies. The injury-induced perturbation of development associated with BPD is also being tackled by using anti-inflammatory approaches, such as interleukin (IL)-10 [42] or inflammatory chemokine blockade [43], and by optimising ventilation strategies [44] to mitigate damage and protect alveolar formation.

CSF-1 provides a particularly attractive candidate for use in this setting because it is a growth factor with proven organogenic [27,28], anti-inflammatory [29,45] and regenerative capabilities [12,46]. During development, endogenous CSF-1 preferentially regulates trophic macrophages associated with organogenesis, as evidenced by the widespread developmental defects observed in CSF-1-deficient mice [20–22]. Functions of trophic macrophages that support organogenesis include apoptotic clearance of cellular debris associated with tissue remodelling [47], regulation of angiogenesis through the production of angiogenic factors [48,49] and by physically directing vascular development [50]. Macrophages act as potent effector cells producing a range of important trophic mediators such as insulin-like growth factor-1 (IGF-1) [51], wingless-type MMTV integration site family, member 7b (Wnt7b) [52], transforming growth factor-β (TGF-β) [53] and MMP9 [54], which are involved in epithelial proliferation and matrix reorganisation. These processes are all essential in lung development, particularly in alveolarisation. Macrophages in both the embryonic lung and the postnatal lung undergoing alveolarisation demonstrate a gene expression profile, indicative of a trophic M2 macrophage activation state [23,27].

The role of CSF-1 in polarising macrophages towards an M2 phenotype may also provide beneficial effects in hyperoxia through its involvement in immune dampening [29]. Inflammation has a negative impact on lung development [55,56]. Inflammatory cell influx and release of pro-inflammatory mediators promotes apoptotic and necrotic cell death that disrupts lung morphogenesis and impairs function. Furthermore, inflammatory activation disrupts organogenic signalling pathways by altering expression of key genes important in lung development [57,58]. While inflammatory challenges such as lipopolysaccharide (LPS) or IL-1β administration in animal models of chorioamnionitis promote lung maturation, the mechanism is distinct from alveolarisation and is instead a survival adaptation that comes at the expense of proper alveolar formation resulting in a lung pathology associated with BPD [59].

The infiltration of inflammatory macrophages is associated with the progression of lung injury and pathology [60,61]. However macrophage diversity is revealing the importance of lung macrophages in both the injury and repair stages [16], and differential activation suggests that a GM-CSF driven M1 pro-inflammatory response may be distinct from CSF-1-mediated stimulation of macrophages to take on a remodelling/anti-inflammatory M2 phenotype [29]. In a recent study, CSF-1 was associated with repair and rescue of alveolar formation following hyperoxia in the mouse [30]. It was reported that the administration of MSC–conditioned media into a neonatal murine hyperoxia model reduced inflammation and prevented alveolar and vascular damage [30]. Interestingly, this correlated with high levels of CSF-1, indicating that the developmentally protective effect of MSCs may be indirectly mediated by the immunomodulatory effect of CSF-1 [30].

In the present study, CSF-1 was administered to a neonatal murine hyperoxia model and growth, lung structure and respiratory function were assessed. In the first treatment regime, CSF-1 was administered post-hyperoxic injury to investigate whether CSF-1 could rescue developmental perturbation and promote alveolar formation. Results demonstrated limited success of CSF-1 in the mitigation of hyperoxia-induced injury. Deficits in growth were not improved, with the body weights of CSF-1-treated mice comparable to the Hyp + PBS group. With regard to lung function, a negative impact was revealed at P11 at the end of treatment. In parameters such as total cycle time, expiration time and minute volume/body weight ratio the detrimental effect of hyperoxia was exacerbated with CSF-1. Interestingly, these initial defects did normalise over time and by P35 no differences between treatment groups were observed. This normalisation was also seen in the Hyp + PBS group, and therefore CSF-1 treatment did not accelerate or enhance functional recovery, although increased numbers of CSF-1R+ macrophages were evident. Nevertheless, it is noteworthy that CSF-1 did not have a negative impact on the parameters measured in the adult. It is interesting that earlier perturbations observed with hyperoxia resolved by adulthood. Whether this impacts on the lungs capacity to cope with challenges and aging in later life is unknown. Furthermore questions remain about potential negative effects associated with catch up growth. Indeed CSF-1R+ macrophages have been shown to be increased in the alveolarisation stage of lung development [23], however whether in this setting the increase in CSF-1R+/F4/80+ macrophages is retained and whether it is associated with positive or negative outcomes in lung physiology is also of ongoing interest.

Oxygen supplementation is a critical life saving intervention for babies with respiratory distress, and clinically any maturation-based therapies would need to be delivered concurrently. Therefore, in the second part of this study, CSF-1 was administered from birth in conjunction with hyperoxic exposure, in a setting where a prophylactic effect of CSF-1 treatment was aimed at preventing damage and alveolar loss and enhancing alveolarisation. A shortened exposure time was utilised to enable the 5 daily doses to be administered in conjunction with hyperoxia. The lack of growth restriction is likely due to the decreased oxygen exposure, as pup litters size was consistent amongst regimes. Structurally and functionally, the lungs of both hyperoxia groups were impacted negatively. Morphometric analysis in the adult revealed significant increases in MLI, with fewer, larger alveoli in both the Hyp + PBS and Hyp + CSF-1 groups compared to Norm. Although CSF-1 was not found to improve the structural alterations caused by hyperoxia, this treatment did not exacerbate the negative impact of hyperoxia and no significant difference between PBS and CSF-1-treated mice was observed. There were no differences between any groups at P5, although this time coincides with the beginning of the alveolarisation phase. Of note is that Hyp + CSF-1 values began to approach Norm values in all parameters. Functionally in the adult, CSF-1 did not exacerbate the adverse effects on lung function that were characteristic of hyperoxia exposure, as represented by the Hyp + PBS group. Although no negative effect of CSF-1 was identified, it must be noted that no functionally beneficial effects with regard to preventing hyperoxic damage or further enhancing lung development were observed. The only changes with CSF-1 at P35 were in tidal volume and minute volume. However, these were normalised when body weight was accounted for and, relative to their size, there were no changes in these parameters as a result of CSF-1 treatment.

Overall, CSF-1 treatment showed little effect in promoting alveolar formation in the hyperoxic settings employed in this study. Ongoing studies examining the role of M2 polarisation in the protection and rescue of hyperoxic injury with be important before a definitive statement that CSF-1-responsive macrophages are not effective can be made. Also further experiments utilizing a premature birth model – where animals are prematurely removed from their high uterine CSF-1 environment – will be important to better model this aspect of the clinical situation. However, in this study the finding that CSF-1 treatment did not exacerbate the pathological response to high oxygen levels was encouraging in that macrophage-mediation may provide a novel strategy for the immunomodulation and trophic promotion of alveolar development. In particular in the second regime where potential negative outcomes were a legitimate concern, CSF-1 did not exacerbate damage with concurrent oxygen supplementation. This is a significant finding that is important for any potential clinical use of CSF-1, and supports ongoing studies to improve understanding of the role of CSF-1 in alveolar formation.
